# The effect of dietary halloysite supplementation on the performance and meat quality of pigs and some air indices in piggery

**DOI:** 10.1038/s41598-022-24987-9

**Published:** 2022-11-29

**Authors:** Małgorzata Nadziakiewicz, Marcin Wojciech Lis, Piotr Micek

**Affiliations:** 1 CIECH Sarzyna S.A, ul. Chemików 1, 37-310 Nowa Sarzyna, Poland; 2 Department of Zoology and Animal Welfare, University of Agriculture in Krakow, Mickiewicza 24/28, 30-059 Krakow, Poland; 3 Department of Animal Nutrition and Biotechnology, and Fisheries, University of Agriculture in Krakow, Al. Mickiewicza 24/28, 30-059 Krakow, Poland

**Keywords:** Biotechnology, Biogeochemistry, Health care

## Abstract

Halloysite, a clay mineral of the kaolin, has specific properties, characteristic for the conditions in which it was formed. The aim of the study was to determine the effect of halloysite from the Dunino deposit in a complete diet for pigs on daily body weight gain (BWG), feed conversion ratio (FCR), blood parameters, chemical composition of meat and chosen environment indices in the piggery. The trial was conducted on 144 piglets from weaning (d 29) to 85th day of life, then on 40 porkers divided into two groups. Animals were fed diets without (group C) or with halloysite (group E; 1.5% until d 128, then 1.0%). Pigs from group E were characterized by a lower number of days with diarrhea. The average BWG in E group was 44 g higher than in group C (P < 0.01). In turn, during the fattening period, the difference in BWG between groups was 60 g in favor of E (P < 0.05) and average FCR in group E was 4.9% lower compared to group C (P < 0.05). At the end of the study, the pigs from group E had 6.9% higher final body weight (P < 0.01). During both the morning and evening measurements, ammonia concentration in the air was lower by 16.3% and 23.8%, respectively. The use of halloysite enabled improvement of pig fattening efficiency, while reducing the costs of pork production and the negative effect of ammonia on the animals' welfare and environment.

## Introduction

Among the many difficulties faced by pig farmers, intestinal disorders and diarrhoea are some of the most serious problems, aggravating the already difficult situation of the pig meat sector in Europe. Previously used antibiotic growth promoters (AGP) were withdrawn from use in the interests of human health and the avoiding the possibility of cross-resistance of humans to therapeutically used products^1^. Another significant problem in many herds of pigs is low fattening efficiency, which negatively impacts the profitability of production. Therefore, improving fattening results may reduce the costs of pork production and thus enhance the economic results of a farm. In this context, the development of new natural origin feed additives for accelerating the achievement of the designated fattening effects, maintaining the optimal composition of the gut microbiota of pigs, and effectively preventing diarrhoea and its negative consequences is highly desirable.

Geophagy has been considered as an adaptive behaviour in humans and animals, and clays have been considered healing materials since ancient times. As geophagia is occasionally a habit of animals, physiologically it is assumed that earthy substances might have some beneficial effects on body function^2^. In the livestock industry, pigs are especially susceptible to *Escherichia coli* infection. This common disease can damage the intestinal barrier and, by causing disorders, reduce the productive capacity of animals. According to the EFSA^3^ report, some clay minerals, such as, e.g., kaolin, have the ability to bind pathogenic microorganisms, in addition to mycotoxins, heavy metals, plant metabolites, poisons, or diarrhoea-causing enterotoxins. Actually, Laurent^4^ has shown that clays added to a pig’s diet bind and absorb diarrhoea-inducing toxins produced by *E. coli* bacteria, without negatively impacting the animals’ growth or digestion. In turn, Toprak et al.^5^ lists a wide range of other applications of clay minerals. These feed additives are used in the feed industry as a pellet binder, simultaneously improving ammonia utilization, binding toxins and heavy metals, and reducing bloat and other metabolic disorders in the gastrointestinal tract (GIT) of animals.

Naturally occurring phyllosilicates, which include clays and clay minerals, are a class of rock-forming minerals with a sheet structure^2^. In animal nutrition, the most promising results to date have been obtained with montmorylonite, smectite, illite, kaolinite, and biotic and zeolite clinoptilolite for their ability to replace AGP and to maintain swine health and performance^6^. Owing to the ability of ion exchange of cations and sorption of organic substances, these minerals show a number of beneficial properties. Generally, the effect of clay adsorbents on the efficiency of animals can be explained by several different mechanisms. It is assumed that clay minerals reduce the speed of feed passage along the intestines, which results in better nutrient conversion and higher water resorption^7^. The results of other authors^8^ confirm the improvement of the digestibility of feed nutrients, thereby increasing protein and energy utilization by animals when clay or clay minerals are used. Another important factor is the environmental aspect of intensive pig production systems producing large amounts of waste which contribute to land, air, or water pollution. In this context, mineral sorbents could play an important role for the whole pig industry as an agent to effectively reduce dangerous emissions^9^.

Kaolin contained in aluminosilicates is used predominately to counteract the negative effects caused by mycotoxins in feeds, e.g., in cereal grains such as maize. Due to the harmfulness of zaerealenone (ZEN), which causes significant negative economic effects on swine production, there is a growing concern that exposure to ZEN during pregnancy affects the health of the offspring due to changes in the development of the immune system^10^. For this reason, halloysite (a phylsilicate in the kaolinite group), has been tested in many trials for the possibility of reducing ZEN toxicity in pig farming^11^.

Halloysite is a tubular mineral of volcanic origin characterized by high porosity and high ion exchange. It does not swell in water, which distinguishes it from other minerals such as, e.g., montmorillonite. There are no two kaolins exhibiting equal properties. It was shown that kaolin properties are influenced by the environment, formation, and deposition conditions. Therefore, the results of studies carried out on kaolin from a certain deposit cannot be extrapolated to any other kaolin deposit^12^. The halloysite used for the present study came from the Dunino deposit (Poland), and stands out for its specific plate-tubular structure with the ratio of halloysite to kaolinite of 66:34. Dunino is one of the largest halloysite deposits in the world. It contains at least 10 million tonnes of homogeneous raw material, which could be a plentiful source of natural supplements for animal nutrition.

We hypothesize that the unique properties of halloysite from the Dunino deposit can improve the efficiency of fattening while at the same time improving the associated parameters, such as nutrient utilization of feed, meat quality, or environmental conditions in pig houses. Fattening and slaughter parameters as well as the physicochemical traits of the meat determine the economic results of pig production. In this context, the content and composition of fats in pork products are one of the most important traits, positively correlated to many other traits of culinary meat quality. The fatty acid composition in pork fat is strictly related to meat tenderness, firmness, and juiciness, as well as to the meat’s taste and its storability. Therefore, this study aimed to determine the effect of halloysite supplementation to the complete diet of pigs on growth performance, carcass characteristics, selected meat quality, and blood parameters, as well as on some air environment indices in piglet pens.

## Materials and methods

### Approval for animal experiment

The animals were treated in a manner according to the principles stated in Directive 2010/63/EU, regarding the protection of animals used for experimental and other scientific purposes, enforced in Poland by Legislative Decrees 266/2015 and 638/2020. Moreover, the practices for animals were: (1) equivalent to practices undertaken for the purposes of recognized animal husbandry; (2) equivalent to practices undertaken for the primary purpose of identification of an animal; and (3) not likely to cause pain, suffering, distress or lasting harm equivalent to, or higher than, that caused by the introduction of a needle in accordance with good veterinary practice, therefore an ethics approval by an institutional review board is not necessary according to Directive 2010/63/EU Art 2.5.e-f (OJ L 276, 0.10.2010, p. 33–79) the Act 2015/266/RP Art.1.2. (O.J. 2015 pos. 266).

The study reported in the manuscript follows the recommendations in the ARRIVE guidelines (*PLoS Biol* 18(7), e3000411. https://doi.org/10.1371/journal.pbio.3000411) and was registered under the number BZ/70/15/WHBZ.

### Experimental design

The trial was conducted at the Pawłowice Experimental Farm, operating under the National Research Institute of Animal Production in Kraków (Poland, 51°49′15.4′′ N 16°44′44.0′′ E). The experiment was conducted in two stages on pig hybrid line 990. On the day of weaning (d 29; the first stage of the experiment), the piglets of polish hybrid line 990 were weighed, randomly assigned into two feeding groups (C and E), and reared to 85 day of life (starter and grower period). Each group consisted of 72 piglets arranged in nine pens with eight piglets per pen (4 barrows and 4 gilts in each pen), 144 in total. On the 85th day of life, 20 porkers from groups C and E (40 in total) were chosen for fattening (the second stage of the experiment), ensuring equal gender proportions and BW of animals in each group. During the fattening period, the animals were kept in individual pens. Before each stage of the experiment, the room and pens were cleaned, washed, and disinfected. Animals were housed in the same piggery on both sides of the feeding corridor. The animals had constant ad libitum access to water and feed throughout the entire period of the experiment, whereby 4 types of nutritionally balanced complete diets (Table 1) were used. Before weaning all piglets had unlimited access to the same prestarter type diet without halloysite addition then based on the age of the pigs, the following types of diets were applied: starter (d 29–d 43), grower (d 44–d 85), fattening I (d 86–d 128) and fattening II (d 129–d 173). In the control (C) diet, the content of energy and nutrients met the German standards of pig nutrition according to BLL^13^. The experimental (E) group received the same diets as were fed in C, but with halloysite supplementation of 1.5% (15 g per 1 kg of diet). Halloysite mineral from Dunino deposit (Dunino Halloysite Mine, Poland) was in a powdered form, naturally free of asbestos, dioxins and heavy metals. Due to the increased intake of diet with age, animals from the fattening II period (group E, d 129–d 173) received a complete mixture with halloysite supplementation of 1.0% (10 g per 1 kg of diet).

**Table 1:** Composition (g/kg) and nutritive value of experimental diets used in pig nutrition after weaning (29 days of life).

Item	Starter, g/kg		Grower, g/kg		Fattening, g/kg			
	(d 29 to 43)		(d 44 to 85)		(d 86 to 128)		(d 129 to 173)	
	C^a^	E^b^	C	E	C	E	C	E
Wheat	304.8	289.8	353.7	338.7	402.0	387.0	381.7	371.7
Barley	300.0	300.0	300.0	300.0	350.0	350.0	400.0	400.0
Maize	100.0	100.0	100.0	100.0	–	–	–	–
Soy protein concentrate	90.0	90.0	–	–	–	–	–	–
Soybean meal	50.0	50.0	160.0	160.0	100.0	100.0	60.0	60.0
Rapeseed meal	–	–	–	–	90.0	90.0	100.0	100.0
Milk replacer (powder)	100.0	100.0	40.0	40.0	–	–	–	–
Bergafat HTL-306	20.0	20.0	10.0	10.0	–	–	–	–
Molasses	–	–	–	–	20.0	20.0	20.0	20.0
Soybean oil	–	–	–	–	10.0	10.0	10.0	10.0
Calcium formate	10.0	10.0	–	–	–	–	–	–
Limestone	–	–	10.0	10.0	10.0	10.0	10.0	10.0
Monocalcium phosphate	10.0	10.0	8.0	8.0	5.3	5.3	5.0	5.0
Sodium chloride	3.0	3.0	4.0	4.0	3.5	3.5	3.5	3.5
l-lysine	4.2	4.2	4.8	4.8	3.1	3.1	3.5	3.5
dl-methionine	0.5	0.5	0.9	0.9	–	–	–	–
l-threonine	1.3	1.3	1.5	1.5	0.5	0.5	0.7	0.7
l-tryptophan	0.2	0.2	0.3	0.3	–	–	–	–
Probiotic (BONVITAL)^c^	0.4	0.4	0.5	0.5	–	–	–	–
Phytobiotic (Bell-Pig)^d^	–	–	0.7	0.7	–	–	–	–
Xylanase (4000-4a11)	0.5	0.5	0.5	0.5	0.5	0.5	0.5	0.5
Phytase (XT-4a1640)	0.1	0.1	0.1	0.1	0.1	0.1	0.1	0.1
Premix vit./min.^e^	5.0	5.0	5.0	5.0	5.0	5.0	5.0	5.0
Halloysite	–	15.0	–	15.0	–	15.0	–	10.0
**Energy value and chemical composition of feeds**								
Dry matter, %	88.6	88.7	88.8	88.7	89.3	89.0	89.2	88.9
Metabolisable energy^f^, MJ/kg	13.4	13.1	13.0	12.7	13.0	12.7	13.0	12.7
Crude ash, %	4.8	5.1	5.3	5.7	5.6	5.9	5.5	5.8
Crude protein, %	19.0	18.6	17.8	17.3	17.6	17.1	16.0	15.6
Ether extract, %	2.9	2.7	4.0	3.8	2.7	2.5	2.8	2.6
NDF, %	12.2	11.5	12.1	11.8	13.6	13.1	15.8	15.2
ADF, %	4.5	4.4	5.4	4.9	6.1	5.8	7.0	6.6
Lysine^g^, g	12.4	12.3	11.9	11.8	9.5	9.5	9.0	8.9
Methionine^g^ + cys, g	6.7	6.6	6.4	6.4	6.0	5.9	5.7	5.7
Threonine^g^, g	8.1	8.0	7.6	7.6	6.2	6.1	5.9	5.8
Tryptophan^g^, g	2.6	2.6	2.5	2.5	2.1	2.1	1.9	1.9
Total calcium^g^, g	7.6	7.6	7.5	7.4	6.4	6.4	6.3	6.2
Total phosphorus^g^, g	6.5	6.4	5.9	5.9	5.7	5.7	5.5	5.4
Sodium^g^, g	1.5	1.5	1.9	1.9	1.8	1.8	1.7	1.6

^a^Control group fed complete mixtures without halloysite.

^b^Experimental group fed complete mixtures with halloysite addition.

^c^Product containing live lactic acid bacteria.

^d^Product containing natural garlic and oregano extracts.

^e^Mineral-vitamin feed additive adapted to the age of the pigs.

^f^Calculated value according to European Table^14^ as a sum of the ME content of components.

^g^Calculated value from the chemical composition of raw feedstuffs.

The fattening was completed on the 174th day of the animals' life (± 2.03 days) and eight animals from both groups (16 in total) were selected randomly, ensuring equal gender proportions and slaughtered in Slaughter Station for Pigs in accordance with Polish and EU regulations. The slaughter was performed by stunning with high-voltage electric shock (voltage 400 V, amperage 1.3A, frequency 50 Hz) followed by exsanguination. The carcasses were chilled for 24 h at 4 °C and then the measurements were performed on the entire carcasses as well as on the right half of the carcasses, which were also assigned for detailed dissection. However, only major cuts are presented in Table 2 due to their importance to breeding practice. From the right half-carcass of 16 animals (8 from groups C and E), representative samples of the loin (*Longissimus dorsi* muscle) were taken for chemical analysis.

**Table 2:** Some slaughter traits of pigs and meat quality parameters of *m. longissimus dorsi.*

Item	Treatment		P-value	SEM
	C^a^	E^b^		
Liveweight at slaughter, kg	109.24	116.75	0.007	1.60
Carcass length, cm	79.50	82.75	0.027	0.85
Dressing percentage, %	79.08	80.13	0.094	0.39
Weight of carcass, kg	86.63	93.73	0.027	1.47
Weight of right half-carcass, kg	41.66	45.30	0.004	0.74
Carcass meat percentage, %	53.46	52.71	0.287	0.64
Weight of right half-carcass, kg	41.66	45.30	0.004	0.74
Weight of tenderloin, kg	0.41	0.43	0.095	0.01
Weight of loin—whole cut, kg	8.37	9.23	0.007	0.18
Weight of skin and loin backfat, kg	1.89	2.31	0.022	0.11
Weight of loin without backfat and skin, kg	6.48	6.92	0.010	0.10
Loin eye area, cm^2^	49.76	51.73	0.173	0.99
Weight of ham—whole cut, kg	10.76	11.66	0.004	0.18
Weight of ham with backfat and skin, kg	1.48	1.64	0.110	0.06
Weight of ham without backfat and skin, kg	8.07	8.73	0.017	0.16
Average backfat thickness, cm	1.65	1.87	0.089	0.09
Weight of meat of primal cuts, kg	22.27	23.84	0.015	0.37
pH_45_	6.30	6.31	0.452	0.04
pH_24_	5.44	5.48	0.170	0.02
Water holding capacity, %	20.47	22.00	0.247	1.06
Meat colour brightness, L*	54.00	53.56	0.358	0.56

^a^Control group fed complete mixtures without halloysite.

^b^Experimental group fed complete mixtures with halloysite addition.

Backfat thickness was measured with a caliper to the nearest 0.1 cm, at five locations: at the thickest point over the shoulder, on the back above the joint between the last thoracic and first lumbar vertebrae, at three points above the loin—above the rostral edge (loin I), above the middle (loin II) and above the caudal edge (loin III) of gluteal muscle section. These measurements were used to calculate the average backfat thickness from five measurements. Next, the half-carcass was dissected into different cuts (Table 2) and weighed. The contour of the *longissimus dorsi* muscle section was traced using a plastic film on the loin at the intersection of the last thoracic vertebra and the first lumbar vertebra, on the cephalic plane. The height and width of the longissimus dorsi muscle was measured on this contour and the loin eye area was planimetered. The loin and ham cuts were dissected into tissues to estimate carcass meat percentage. The meat content of primal cuts was expressed as a percentage of chilled carcass weight.

### Production indices

On the last day of each period (d 43, 85, 128, 173), all pigs were weighed individually (BW) and on this basis, daily weight gain (BWG) was calculated. During the entire period of the experiment, no animal losses were recorded. Feed intake (FI) was monitored for each pen separately until d 85 (starter and grower period) and then individually for each animal during the fattening period. Feed conversion ratio (FCR) was calculated for each period and the entire cycle according to the formula: FCR = FI (kg)/BWG (kg).

### Blood parameters

On the day preceding the end of the experiment and slaughter, blood was collected from the external jugular vein into a tube containing an anticoagulant (disodium EDTA, 15 mg/10 ml blood). Plasma was recovered by centrifugation at 6500× for 30 min using an iFuge D06 PE (Neuation Technologies Pvt. India). The concentration of triglycerides (TG; Liquick Cor-TG30, No 2-262), total cholesterol (TCH; Liquick Cor-CHOL60, No 2-204), high-density lipoprotein (HDL; Liquick Cor-HDL, No 2-053) and low-density lipoprotein (LDL; DIRECT 500, No 2-194) fractions of cholesterol in plasma were determined by a colorimetric method using Cormay (Lublin, Poland) monotests. Absorption was recorded using a Metertech SP-830 Plus (Merazet, Poznań, Poland) spectrophotometer.

### Environmental parameters

Environmental conditions in the piglet pens, such as: temperature, relative air humidity, and the concentration of carbon dioxide (CO_2_) and ammonia (NH_3_) in the air, were measured twice a day (at 0700 h and 2000 h—before and after finishing the staff work in piggery). Measurements were taken at d 84 and 85 of the piglet’s life (end of the grower period) from 18 pens (9 per group), always at the height of the animals' heads. The temperature and relative humidity of the air were measured using an electronic TA465 meter (Test-Therm Ltd. Poland). The concentration of CO_2_ and NH_3_ was tested using the Polytector II gas meter No. G750 (GfG, Dortmund, Germany). No presence of hydrogen sulfide (H_2_S) was found in the piggery above the threshold sensitivity of this apparatus.

### Chemical analysis of samples and meat quality parameters

Prior to chemical analysis, air-dried samples of the diet were ground to pass through a 0.75 mm sieve with a Pulverisette 15 Laboratory Cutting Mill (Fritsh GMBH, Idar Oberstein, Germany) and analysed to determine the content of dry matter (DM), crude protein (CP), and ether extract (EE) using standard analytical procedures (procedures no. 934.01, 976.05 and 920.39, respectively; AOAC^15^. Neutral detergent fibre determined with heat-stable amylase (aNDF) and acid detergent fibre (ADF^15^; (Official Method 973.18) were detected using an Ankom^220^ Fiber Analyzer (Ankom Technology, USA). The same procedures were used for DM and CP determination in faeces collected at d 84 and 85 of piglet’s life from 18 pens (9 per group).

Samples of approx. 150–200 g of meat were collected from *m. longissimus dorsi* over the last 3 thoracic vertebrae to determine DM, CP^15^ (Official Method 934.01 and 976.05, respectively), and cholesterol content by gas chromatography (TRACE GC ULTRA apparatus; Thermo Scientific, Milan, Italy) with an HP-5 column, using the method of direct saponification for the sample preparation according to AOAC Official Method 994.10-1994. Additionally, the following indicators of meat quality were studied: intramuscular fat (IMF), loin brightness (L*), water holding capacity, and meat pH 45 min (pH_45_) and 24 h (pH_24_) postmortem. The content of IMF was determined as crude fat by Soxhlet extraction^15^ (Official Method 991.36) with fat solvents (Soxtherm 406, Gerhardt, Germany). Loin brightness was measured on the loin surface at 24 h postmortem using a Minolta CR 310. Meat pH was estimated using a pH Meter PCE-PH20M (PCE Instruments, Southampton, England). Water holding capacity was determined by the Grau and Hamm method^16^. To analyse the composition of fatty acids (FA), meat samples of loin were collected and immediately preserved and stored at –20 °C until analysis. Meat FA profile was determined by gas chromatography with a TRACE GC ULTRA apparatus, equipped with a 30 m capillary column SUPELCOWAX 10 (Sigma-Aldrich Co. LLC) of 0.25 mm inner diameter following to Żak et al.^16^.

### Statistical analysis

Data were analyzed as a completely randomized design using the GLM procedure of SAS (ver. 9.2; SAS Inst. Inc., Cary, NC). Before analysis, normal distribution of the data was verified using PROC UNIVARIATE in SAS, and no data transformation was necessary. The statistical model included the random effect of pig and the fixed effect of treatment (halloysite addition). Furthermore, the effect of sex and its interaction with diet was tested in the model; however, it turned out not to be significant (P > 0.05) and was removed from the model. Data on piglet FCR (starter and grower period) as well as for DM and CP content in faeces were analysed as a randomized complete block design, in which the pen was considered a block (n = 18) and the mean value of 8 animals, the experimental unit. Due to the deficiency of one indicator of meat quality parameters as well as removed from statistical analyses weight gain for one animal in fattening period (outliers), the results are presented as least square means and their corresponding standard errors. Significance was declared at P ≤ 0.05, and tendencies were discussed when 0.05 ≤ P ≤ 0.10.

## Results

Incorporation of halloysite to the diets of group E occurred in exchange for the reduction of the share of wheat grain in the mixtures. As an effect, compared to the C diet, a slight increase of crude ash and a decrease of organic components and metabolisable energy concentration was observed (Table 1). During the entire cycle of production, a total of 6 diarrhoea days were recorded in group C, while 3 diarrhoea days were recorded in group E (data not shown in table). The initial BW of piglets on the day of weaning (d 29) did not differ between treatments (Table 3). On d 42, piglets from group E had higher BW by 0.30 kg (P < 0.05) and on d 85 by 2.45 kg (P < 0.01). At the end of the fattening period, the pigs from group E had 6.9% higher final BW (P < 0.01) compared to C (117.01 vs. 109.5 kg, respectively). For the period of rearing (d 29 to 85), the average BWG in group E was 44 g higher (P < 0.01) than in C. In turn, during the fattening period (d 86 to d 173), the difference in BWG between treatments was 60 g (P < 0.05) in favour of E (+ 7.1%). Regardless of the period, animals from group E were characterized by better feed utilization (Table 3). Between d 29 and d 85, the average FCR in group E was 1.69, which was 7.1% lower compared to group C (P < 0.01). Average FCR in group E during fattening (d 86 to d 173) was 4.9% lower compared to C (P < 0.05). That means saving an average 0.13 kg of feed per kg of BWG.

**Table 3:** Effect of dietary halloysite supplementation on production indices of pigs after weaning and dry matter and crude protein concentration in the faeces of piglets.

Item	Day of life	Treatment		P-value	SEM
		C^a^	E^b^		
Body weight, kg	29	7.46	7.60	0.436	0.09
	43	9.52	9.82	0.014	0.11
	85	32.42	34.87	0.001	0.41
	173	109.50	117.01	0.001	1.06
Daily body weight gain, g	29–43	142	164	0.014	4.32
	44–85	545	596	0.003	8.49
	29–85	444	488	0.001	6.82
	86–173	842	902	0.038	11.03
Feed conversion ratio, kg/kg	29–85	1.82	1.69	0.008	0.02
	86–173	2.68	2.55	0.023	0.15
**Faeces**					
Dry matter, %	84–85	25.62	24.61	0.068	0.55
Crude protein, % DM	84–85	22.52	22.83	0.125	0.41

^a^Control group fed complete mixtures without halloysite.

^b^Experimental group fed complete mixtures with halloysite addition.

### Slaughter traits, meat quality, and blood parameters

The animals from both feeding groups were slaughtered at the same age (± d 2.03), but with different liveweight, which impacted differences in the weight of carcasses and individual cuts (Table 2). Fatteners from group E were characterized by higher carcass length, carcass weight, and right half-carcass weight (P < 0.05), and tended to higher dressing percentage (P = 0.094). Carcass meat percentage was unaffected by the treatment (P > 0.05), as were meat quality parameters (pH, water holding capacity, and loin meat brightness). Weight of loin and weight of ham without backfat and skin were higher in group E compared to C (P < 0.01); however, animals from group E tended to have higher average backfat by a thickness of 5 measurement points (P = 0.089). Except for the tendency for higher TCH concentration in loin in group E (P = 0.072), no effect of treatment was found on DM, CP, or IMF concentration in this meat (Table 4). Regarding blood plasma, animals from group E had higher concentration of TCH (P < 0.05), without any differences from group C in HDL, LDL, or TG. Apart from the lower proportion of C20 FA in the sum of FA of the loin fat from group E (P < 0.05), no other differences were found in FA profile between treatments (Table 5). Similarly, there were no differences between treatments in the sum of unsaturated fatty acids (UFA), monounsaturated fatty acids (MUFA), polyunsaturated fatty acids (PUFA) or in the proportion of fatty acids from n-3 (N3FA) to n-6 (N6FA) FA.

**Table 4:** Chemical composition of raw loin and blood lipid profile of pigs.

Item	Treatment		P-value	SEM
	C^a^	E^b^		
**Loin**				
Dry matter, %	25.29	25.84	0.456	0.21
Crude protein, % DM	87.43	87.70	0.369	0.59
IMF^c^, % DM	4.45	4.46	0.496	0.40
Cholesterol, mg DM	231.73	248.72	0.072	6.49
**Blood plasma, mg/dl**				
HDL	35.02	37.36	0.326	2.20
LDL	49.85	49.66	0.247	3.33
Cholesterol	100.53	104.82	0.035	3.67
Triglicerides	59.41	60.44	0.494	3.45

^a^Control group fed complete mixtures without halloysite.

^b^Experimental group fed complete mixtures with halloysite addition.

^c^Intramuscular fat.

**Table 5:** Fatty acid composition (%) of intramuscular fat of *m. longissimus dorsi.*

Fatty acid	Treatment		P-value	SEM
	C^a^	E^b^		
C10	0.43	0.48	0.296	0.04
C12	1.10	1.17	0.346	0.09
C14	4.41	4.50	0.425	0.22
C15	0.35	0.33	0.362	0.03
C16	29.10	28.80	0.324	0.31
C16:1 n7	3.65	3.66	0.488	0.17
C17	0.50	0.48	0.188	0.01
C18	9.65	9.73	0.457	0.36
C18:1 n9	35.20	35.60	0.378	0.61
C18:2 n6	9.87	9.07	0.273	0.63
C20	0.22	0.17	0.047	0.02
C18:3 n6	0.05	0.04	0.338	0.00
C20:1 n9	0.36	0.35	0.441	0.02
C18:3 n3	1.33	1.62	0.193	0.16
C20:2 n6	0.12	0.11	0.156	0.01
C20:4 n6	1.01	0.99	0.434	0.07
C20:5 n3	0.11	0.11	0.432	0.01
C22:6 n3	0.11	0.09	0.105	0.01
Others	2.44	2.69	0.134	0.14
SFA^c^	45.76	45.66	0.472	0.64
MUFA^d^	39.20	39.61	0.395	0.73
PUFA^e^	12.60	12.03	0.358	0.74
UFA^f^	51.80	51.65	0.454	0.64
N3FA^g^	1.55	1.83	0.211	0.16
N6FA^h^	11.05	10.21	0.274	0.66

^a^Control group fed complete mixtures without halloysite.

^b^Experimental group fed complete mixtures with halloysite addition.

^c^Saturated fatty acids.

^d^Monounsaturated fatty acids.

^e^Polyunsaturated fatty acids.

^f^Unsaturated fatty acids.

^g^N − 3 fatty acids.

^h^N − 6 fatty acids.

### Environmental parameters

Differences were found between treatments (P < 0.01) in air temperature at the height of the animals' heads (Table 6). However, the obtained values were not consistent between the morning and evening measurements—lower in the morning and higher in the evening for group E compared to C (Table 6). Relative humidity (P < 0.01) as well as CO_2_ concentration (P < 0.05) in the air differed between groups only during evening measurements, whereas in both cases, the values were lower for group E compared to C (by 11.0% and 3.8%, respectively). In turn, lower NH_3_ concentration in the air was found during both the morning and evening measurements (P < 0.01) in pens from group E compared to C, by 16.3% and 23.8%, respectively.

**Table 6:** The quality of the air environment in the piglet pens.

Item	Morning				Evening			
	C^a^	E^b^	P-value	SEM	C	E	P-value	SEM
Temperature, °C	21.02	20.68	0.006	0.07	20.01	21.09	0.001	0.14
Relative humidity, %	68.79	68.71	0.471	0.56	61.7	54.93	0.001	0.96
CO_2_, ppm	3575	3535	0.348	49.94	3318.33	3191.67	0.037	35.53
NH_3_, ppm	38.89	32.56	0.001	0.94	20.56	15.67	0.001	0.59

^a^Control group fed complete mixtures without halloysite.

^b^Experimental group fed complete mixtures with halloysite addition.

## Discussion

Feedstuff is an integral part of the food chain and plays important roles in the productivity of farm animals as well as in the composition, safety, and quality of livestock products. The search for ways to achieve better economic results in meat production while meeting higher consumer expectations causes a growing interest in natural feed additives which will improve BWG or FCR in pigs. Thus far, benefits in animal productivity from the use of clay or clay minerals have been noted for both weanling pigs and growing-finishing pigs^17^.

One of the most studied clay minerals used as a feed additive for pigs is clinoptilolite (CPL). In the trial of Defang and Nikishov^18^, CPL was used in diets for fatteners at levels of 3.0%, 4.0%, or 5.0%. Achieved BWG in all experimental groups was higher compared to the control; however, the best result occurred in the group with 4% of CPL addition. In this case, BWG increased by 9.4%, FCR was reduced by 8.5%, and a longer carcass was observed. In turn, when CPL was used by Prvulovic et al.^19^ at the proportion of 0.5%, only 1.2% improvement in BWG and 0.4% reduction in FCR resulted for the whole cycle of production. Interestingly, during the last 45 days of fattening, an inverse effect was noted (i.e., more favourable indicators for the control group). In this experiment, significantly lower TCH and higher TG concentration in blood were also observed in the treatment group.

The data described above are in line with the results of our study, in which at first 1.5% then 1.0% additions of halloysite were used. In the second phase of fattening, the favourable difference in the increment of BWG narrowed, but remained higher in group E, compared to C. It can be speculated that the use of minerals such as CPL or halloysite has a positive effect on nutrient digestibility, resulting in higher BWG and lower FCR indices. In our research, these observations have been confirmed by the higher performance of animals, including higher liveweight at slaughter, weight of carcass, or carcass long. In group E, higher meat weight of primal cuts, including the weight of ham or loin, was caused mainly by higher liveweight at slaughter. These observations are partially in line with the findings of Sardi et al.^20^, who did not observe an improvement in growing parameters (BWG, FCR) of fatteners fed with CPL but noted a higher percentage of primal cuts and improvement in their lean-to-fat ratio. It is also worth noting that in our study dressing percentage, carcass meat percentage, and average backfat thickness remained unaffected by treatment, which may indicate an equal increase in protein and energy utilization of animals from group E. As a consequence, there were no differences in the meatiness or fat content of the carcasses. However, to achieve the same final BW in group C, as was in group E, the fattening would have to be extended by 20 days with the consumption of an additional 46.9 kg of feed per animal. This result, combined with the low cost of clay minerals as feed additives, may be an economic argument for the use of halloysite in the nutrition of fattened pigs.

In contrast to the findings of Prvulovic et al.^19^, other researchers^6^ observed a slight increase in TCH concentration in the blood of pigs fed with CPL, without affecting TG levels. This could mean that the influence of clay additives on the blood lipid profile of pigs is not clear or fully proven. Actually, during the last few decades, increasing attention has been paid to the link between the meat quality of fattened pigs and the lipid profile of their blood. Triacylglycerols play the role of high-energy fuel in the animal body or are deposited as a reserve material in the form of adipose tissue^21^. The concentration of cholesterol as well as its HDL and LDL lipoprotein fractions in the blood depends mainly on genetic factors such as race or sex, but some environmental factors such as breeding, nutrition or endogenous production of these compounds in the liver could also modify their concentration in blood and meat. In our study, the use of halloysite caused an increased in TCH concentration in blood plasma, without affecting other lipid profile indices. As a consequence, a tendency to higher TCH concentration in loin meat was observed. However, the improvements discussed above in nutrient conversion in pigs fed halloysite did not translate into any changes in the chemical composition of meat or the fatty acid profile of intramuscular fat.

According to Laurent^4^, clay minerals have the ability to bind pathogenic microorganisms and diarrhoea-causing enterotoxins, maintaining the optimal composition of the gut microbiota of pigs. Observed in our research, the tendency toward higher DM content in faeces as well as favourable performance indices in group E seems to confirm this finding. Furthermore, it allows us to suppose that halloysite efficiently reduced the speed of feed passage along the intestines, which resulted in better nutrient conversion and higher water resorption. Consequently, a reduced number of days with diarrhoea was observed in piglets, which could contribute to the improvement of animal performance during fattening. Without a doubt, dietary clays alleviate the diarrhoea of weaned pigs, but the specific mode of action of these substances in the GUT of pigs remains unclear, especially if any symptoms of diarrhoea or other gut dysfunction were not observed in the control group. In this context, it is worth underlining that in our research a significant reduction in air NH_3_ concentration was observed in the case of group E, both in the morning and in the evening. One of the most likely explanations for this improvement is the fact that halloysite increases nutrient digestibility and N retention. Clays reduce the speed of passage of feed along the digestive tract, which allows more time for digestion^17^, but other halloysite properties, i.e., the binding capacity of harmful gases, are not irrelevant. Such NH_3_ reduction in the air environment is essential both for the welfare of the animals and workers operating the piggery.

Song et al.^22^ achieved a reduction in diarrhoea symptoms caused by enterotoxigenic *E. coli*, as indicated by reductions in both diarrhoea scores and the frequency of diarrhoea, with the use of smectite, kaolinite and zeolite. Moreover, Papaioannou et al.^23^ showed the ability of CPL to reduce the postweaning diarrhoea of piglets. Therefore, it seems that clay minerals have a potential to detoxify the organism and prevent diarrheal diseases. Due to their adsorption and detoxifying capabilities, these minerals appear to be effective agents for the enhancement of piglet growth in the crucial period of weaning and suitable alternatives to antibiotics. However, they are not always effective. According to Bederska-Łojewska and Pieszka^24^, postpartum and the time associated with severe stress caused by weaning and changing nutrition to a solid feed mixture are the periods of the greatest occurrence of diarrhoea. These authors suggest that intense adverse changes in piglets may lead to increased susceptibility to pathogen infections. Therefore, they used kaolinite supplementation (0.6%) in the nutrition of weaned piglets and unexpectedly, an increase in *E. coli* bacteria in the treatment group was noted.

Another frequently studied mineral is montmorillonite (MMT). Duan et al.^25^ tested MMT in pig diets at levels of 0.5%, 1.0%, 2.5%, and 5.0%. In all treatment groups, BWG was reduced by 6.2–14.6%, and no health benefits were observed; therefore, this additive was considered economically unjustified. In turn, Liu et al.^26^ showed that the use of MMT at the level of 0.2% caused a slight increase in BWG and in the feed intake of weaned piglets. Generally, the weaning period is the most important event in the life of pigs and the weaning process is often associated with microbial imbalance and intestinal barrier dysfunction, which is responsible for the stunted growth and diarrhoea observed in the first 2 weeks after weaning. Therefore, in piglet rearing, dietary zinc (Zn) supplementation has commonly been used to relieve postweaning diarrhoea and improve performance. However, this strategy has been criticized as animals excreted large amounts of Zn, which was an environmental hazard. Jiao et al.^27^ conducted a study on the use of MMT prepared by Zn^2+^ ion exchange in completed feed to reduce Zn supplementation in sustainable pig production. It was shown that this MMT increased growth efficiency, intestinal villus height, and crypt depth, and alleviated weaning diarrhoea, as well as improving the intestinal microflora balance and the barrier function of weaned piglets. Therefore, the results of the literature on the use of MMT in pig nutrition are not clear or steady.

The elimination of zinc oxide (ZnO) from the piglets’ diet was also the aim of Tang et al.’s^28^ study. They used polygorskite (PAL) dietary supplementation of 1.8%, 2.4%, or 3.0% instead of ZnO, which resulted in an increase in BWG and nutrient digestibility. At the same time, a decrease in FCR, diarrhoea rate and Zn excretion in faeces in all treatment groups was observed. According to the authors, PAL can be considered an environmentally friendly antidiarrheal feed additive. Similar conclusions were drawn from the research carried out by Zhang et al.^29^ in which 0.2% or 0.3% supplementation of PAL was used.

In summary, it is worth emphasizing that in the literature^2,30^ conflicts are often reported between the results of experiments with the use of clay minerals as feed additives. Some of them describe improvement in BWG or FCR indices, while some show negligible or negative changes. In our opinion, differences between trials may result from different conditions of the experiments, different clay minerals used in the research, or differences between deposits of the same mineral. Indeed, minerals are characterised by various properties due to the varied conditions in which they have developed. According to Sardi et al.^20^, the properties of clays may be related to the deposit and result from the presence of specific compounds or impurities (i.e., feldspar, apatite, calcite). These differences may, to a greater or lesser extent, affect the results of the experiments. However, most results of research show a beneficial effect of dietary supplementation of clay minerals on livestock performance and the economic effects of animal production.

In conclusion, pigs fed a complete diet with the addition of halloysite from the Dunino deposit were characterised by fewer days with diarrhoea and higher growing performance compared to the control group. Significantly higher daily body weight gain and weight of loin and ham cuts in carcasses were accompanied by a lower feed conversion ratio and more favourable environmental conditions in the piglet house. By decreasing the concentration of ammonia in the piggery, halloysite improves the animals' welfare and the working conditions of the farm staff, while reducing the extent to which the farm harms the environment. However, additional research is required to characterize the effect of dietary supplementation of halloysite on the gut microbiota and the suitability of meat for processing.

## Data Availability

The datasets used and/or analysed during the current study available from the corresponding author on request.
